# The Association of Psoriasis, Diabetes Mellitus, and Hypertension: A Meta-Analysis

**DOI:** 10.7759/cureus.48855

**Published:** 2023-11-15

**Authors:** Hyder Mirghani, Abdulaziz Altemani, Ethar Alsaedi, Rahaf Aldawish, Mohammed Alharbi, Reema Alzahrani, Saleh Alatawi, Sarah Altemani, Ahmed H Alanazi

**Affiliations:** 1 Internal Medicine Department, Faculty of Medicine, University of Tabuk, Tabuk, SAU; 2 Dermatology Department, King Fahad Specialist Hospital, Ministry of Health, Tabuk, SAU; 3 Dermatology Department, King Faisal Hospital, Ministry of Health, Makkah, SAU; 4 Dermatology Department, College of Medicine, Sulaiman Alrajhi University, Qassim, SAU; 5 Family Medicine Department, Waerh Primary Healthcare Center, Ministry of Health, Madinah, SAU; 6 Dermatology Department, Faculty of Medicine, King Abdulaziz University, Jeddah, SAU; 7 Dermatology Department, Faculty of Medicine, University of Tabuk, Tabuk, SAU; 8 Internal Medicine Department, King Salman Armed Forces Hospital, Ministry of Defense, Tabuk, SAU

**Keywords:** psoriasis, diabetes mellitus, risk factor, meta-analysis, hypertension

## Abstract

Psoriasis is a systemic disease affecting various organs; however, it is usually thought of as a skin disease. A multidisciplinary approach is needed for better outcomes. The current meta-analysis assessed the association between diabetes mellitus, high blood pressure, and psoriasis. We searched four databases, including Cochrane Library, PubMed, MEDLINE, and Google Scholar, for relevant articles using the following keywords: psoriasis, hypertension, high blood pressure, cardiovascular risk factors, and diabetes mellitus. The author's name, year, and country of publication, diabetes, and hypertension among patients with psoriasis and control subjects were collected and entered into a Microsoft Excel sheet. Out of 1209 articles retrieved, 903 articles remained after duplication removal. From the 82 full texts screened, only seven studies fulfilled the inclusion and exclusion criteria. Psoriasis was associated with diabetes and hypertension: odds ratio 1.38, 95% CI 1.17-1.64; P-value 0.0002, chi-square 224.93, and odds ratio 1.60, 95% CI 1.41-1.81, P-value 0.00001, chi-square 226.59, respectively. Substantial heterogeneity was observed (*I^2^* for heterogeneity, 97%, P < 0.001). A broad approach is needed to address the associated comorbidities and select the appropriate therapeutic approach. Randomized controlled trials investigating the best drugs for the treatment of psoriasis and its associated cardiovascular risk factors are needed.

## Introduction and background

Psoriasis is a chronic inflammatory disease with a strong genetic predisposition; the prevalence varies according to the location, with the highest prevalence observed in Scandinavian regions (11%) and the lowest in some African and Asian countries [1]. There are many clinical subtypes (plaque psoriasis, guttate psoriasis, inverse psoriasis, pustular psoriasis, erythrodermic psoriasis, nail psoriasis, and scalp psoriasis) with plaque psoriasis (psoriasis vulgaris) being the most common. Other variants include pustular psoriasis, which can be localized or generalized, and inverse psoriasis involving skin folds [2]. Psoriasis affects the skin, joints, oral cavity, scalp, and nails. In addition, it is associated with various disorders, including psychiatric disease, cardiometabolic issues, and streptococcal infections. Other factors include smoking, alcohol consumption, and obesity [3]. Psoriasis is considered an immune-mediated systemic disease and is associated with various comorbidities, significantly impacting patients, communities, and healthcare systems [4]. Genetic predisposition and environmental triggers lead to a chronic proinflammatory process, releasing interleukins, tumor necrosis factor, and interferons. These cytokines initiate a vicious cycle of chronic inflammation and keratinocyte hyperproliferation [5]. Targeting the inflammatory pathways and cytokines contributing to the disease pathogenesis can revolutionize psoriasis treatment [6]. Importantly, psoriasis shares its pathogenesis with various comorbidities, including diabetes, myocardial infarction, metabolic-associated fatty liver disease, dyslipidemia, and hypertension [7-8]. However, there is a lack of addressing these dangerous, associated comorbidities.

Psoriasis has been associated with an increased risk of developing other health conditions, including hypertension (HT) and diabetes mellitus (DM). Chronic inflammation in psoriasis can contribute to the development of these conditions. Studies have shown that individuals with psoriasis are at a higher risk of developing hypertension [4]. The chronic inflammation associated with psoriasis may contribute to endothelial dysfunction and arterial stiffness, which are risk factors for hypertension. Psoriasis has also been linked to an increased risk of developing type 2 diabetes mellitus. It can also lead to insulin resistance, a key factor in the development of type 2 diabetes [8].

It is evident that the association between psoriasis, diabetes mellitus, and hypertension presents a significant challenge in the field of healthcare. The complex interplay of these conditions requires a comprehensive approach to diagnosis and management. Furthermore, the potential impact on patient outcomes and quality of life necessitates further research and collaboration among healthcare professionals. Addressing this challenge will require a multidisciplinary approach and a deeper understanding of the underlying mechanisms linking these conditions. Therefore, this meta-analysis aimed to assess the relationship between psoriasis, diabetes, hyperlipidemia, hypertension, and obesity.

## Review

Materials and methods

Eligibility Criteria According to PICOS (Population, Intervention, Comparison, Outcome, Study)

This meta-analysis included retrospective and prospective cohorts, cross-sectional, and case-control studies, evaluating the association between psoriasis, diabetes, and hypertension. Case reports, case series, and studies on animals were not included.

Outcome Measures

The association of psoriasis with metabolic syndrome.

Literature Search and Data Extraction

Two reviewers searched three databases (PubMed MEDLINE, the first 100 articles in Google Scholar, and Cochrane Library) from inception up to May 2023. The keywords psoriasis, hypertension, high blood pressure, and diabetes mellitus were used. In addition, the references of the included studies were searched for relevant articles. We identified 1209 studies and 903 stands after the removal of duplication, from them, 82 full texts were screened and only seven studies were included in the final meta-analysis. A datasheet was used to extract the author's name year and country of publication, diabetes, and hypertension among patients with psoriasis and control subjects.

Risk of Bias Assessment

The Newcastle Ottawa Scale was used to assess the quality of the included studies.

Statistical Analysis

The RevMan system (The Cochrane Collaboration, revman.cochrane.org) was used for data analysis and the DerSimonian and Laird method was applied. Seven studies were pooled, five retrospective and two cross-sectional studies. Dichotomous data were entered manually and random effects were applied due to the substantial heterogeneity. A P-value of <0.05 was considered significant.

Results

**Figure 1:**
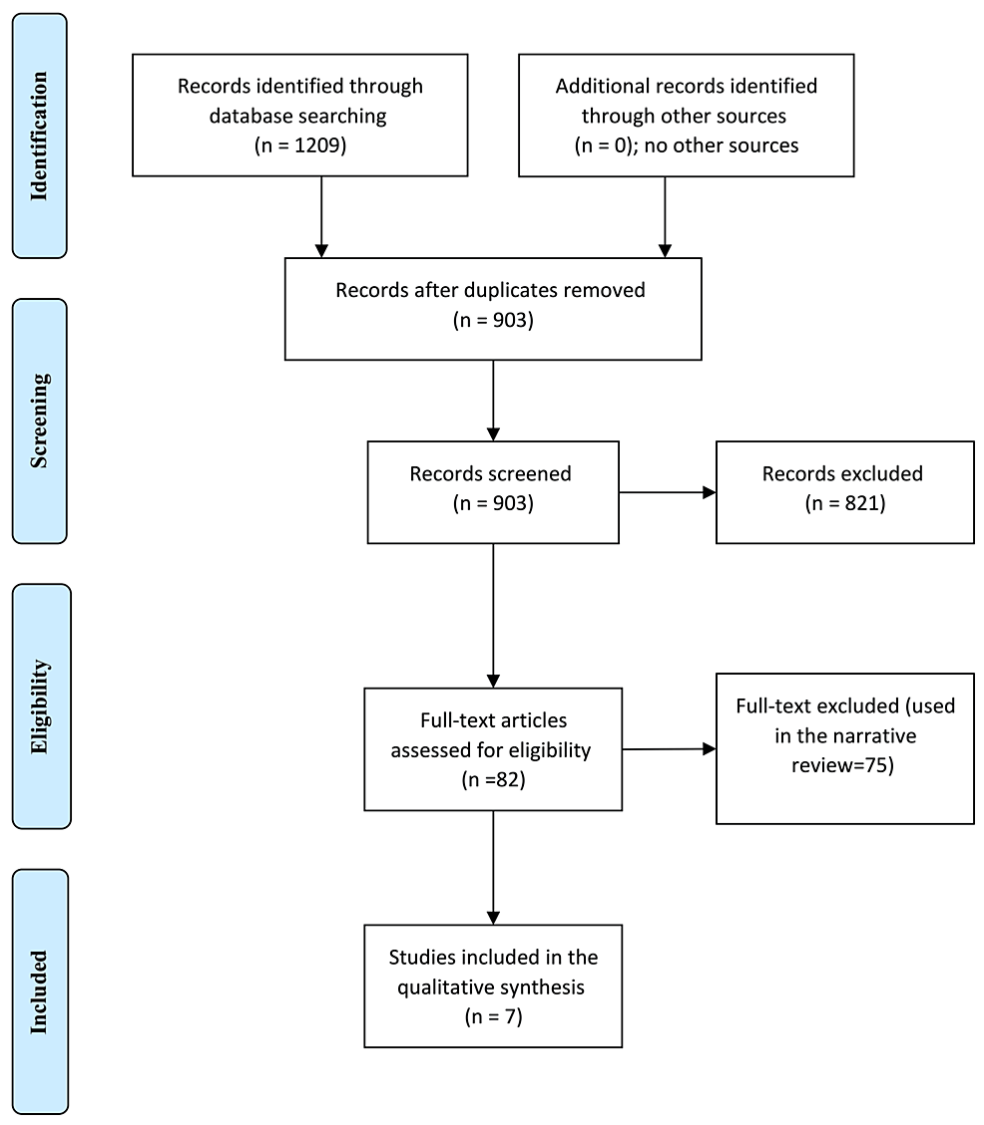
PRISMA Chart of the Included Studies PRISMA: Preferred Reporting Items for Systematic Reviews and Meta-Analyses

The total number of the articles retrieved was 1209; 903 remained after the removal of duplication, of them, 82 full texts were screened. Only seven studies fulfilled the inclusion and exclusion criteria. All the included studies were of good quality according to the Newcastle Ottawa Scale. Five of the included studies were retrospective and two were cross-sectional (Figure 1).

Four of the studies were published in the USA, two in Europe, and one in Asia. The age of the participants ranged from 42 to 55 years, 44.7%-55.6% were women, and patients with psoriasis had more comorbidities (Tables 1-4).

**Table 1:** Diabetes Mellitus Among Patients With Psoriasis and Control Subjects (Data Are Presented As Percentages)

Author	Methods	Psoriasis	Controls	Results
Feldman et al. [9]	Retrospective	17796/114824	14738/114824	Significant
Fernández-Armenteros et al. [10]	Cross-sectional	954/6868	29503/398701	Significant
Feldman et al. [11]	Retrospective	196/1230	123/1230	Significant
Sun et al. [12]	Retrospective	45/307	47/613	Significant
Kaine et al. [13]	Retrospective	2,365/14,898	4,452/35,037	Significant
Kampe et al. [14]	Cross-sectional	1,090/7249	7,503/72,490	Significant
Lee et al. [15]	Retrospective	146/7245	227/7245	Significant

**Table 2:** Basic Characteristics of Patients With Psoriasis and Control Subjects (Data Are Presented As Percentages and Mean ± SD)

Author	Country	Age/ years	Sex/females	Comments
Feldman et al. [9]	USA	53 vs. 53	53.8% vs. 35.8%	Patients had more comorbidities
Fernández-Armenteros et al. [10]	Spain	42.34 years	49.3% vs. 44.7%	More comorbidities among patients compared to controls.
Feldman et al. [11]	USA	48.46 ± 10.75 vs. 48.46 ± 10.75	49.7 5 vs. 49.7%	Patients had more lung, liver, and rheumatic disease
Sun et al. [12]	China	-	-	More comorbidities among patients compared to controls.
Kaine et al. [13]	USA	53.4 vs. 54.8	55.4% vs. 55.6%	Patients were younger and had more comorbidities
Kampe et al. [14]	Slovak	51.5±14.0	51.1%	More comorbidities among patients
Lee et al. [15]	USA	48.1 ± 1.3 vs. 48.1 ± 1.3	51.8% vs. 51.8%	Patients had more comorbidities

**Table 3:** Hypertension Among Patients With Psoriasis and Control Subjects (Data Are Presented As Percentages)

Author	Methods	Psoriasis	Controls	Results
Feldman et al. [9]	Retrospective	45637/114824	38752/114824	Significant, USA
Fernández-Armenteros et al. [10]	Cross-sectional	2143/6868	75753/398701	Significant, Spain
Feldman et al. [11]	Retrospective	440/1230	289/1230	Significant, USA
Sun et al. [12]	Retrospective	141/307	169/613	Significant, China
Kaine et al. [13]	Retrospective	6,297/14898	12,379/35,037	Significant, USA
Kampe et al. [14]	Cross-sectional	2762/7249	21147/72,490	Significant, Slovak
Lee et al. [15]	Retrospective	617/7245	390/7245	Significant, USA

**Table 4:** Quality Assessment of the Included Studies (Quality was Calculated From the Maximum Score of 9)

Reference	Selection	Compatibility	Outcome	Overall
Feldman et al. [9]	3	2	2	7
Fernández-Armenteros et al. [10]	4	1	2	7
Feldman et al. [11]	3	2	2	8
Sun et al. [12]	4	2	2	8
Kaine et al. [13]	3	2	2	7
Kampe et al. [14]	3	2	2	7
Lee et al. [15]	3	2	2	7

**Figure 2:**
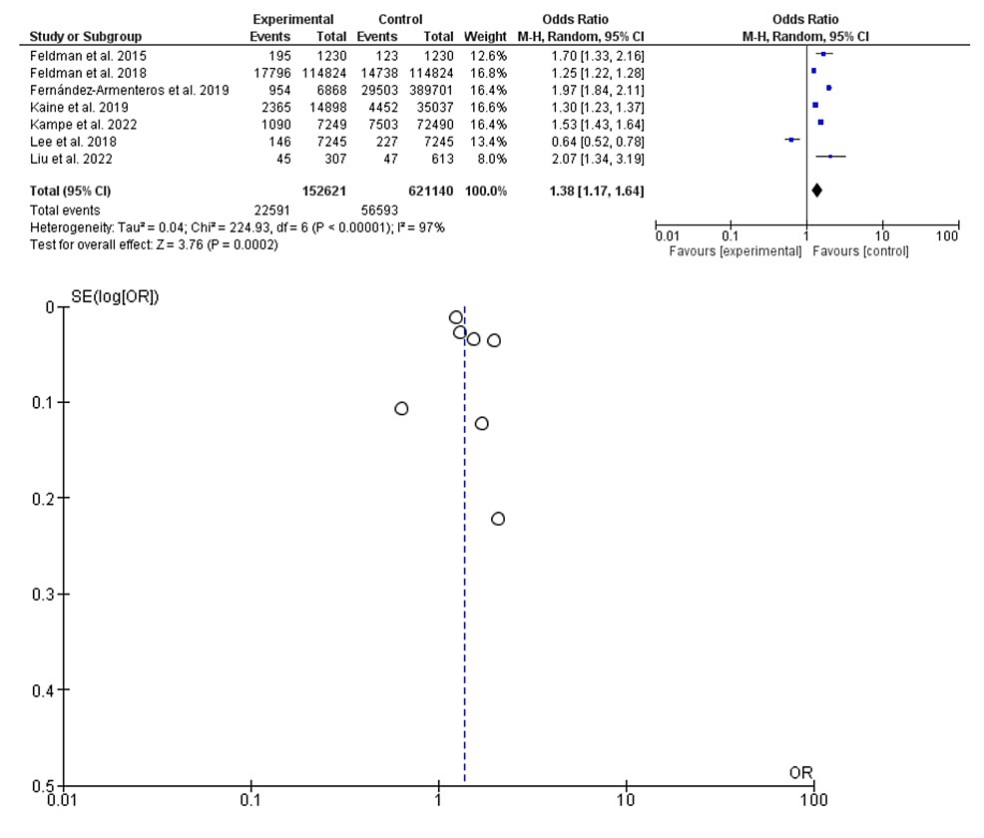
Diabetes Mellitus Among Patients With Psoriasis and Control Subjects Feldman et al. [9]; Fernández-Armenteros et al. [10]; Feldman et al. [11]; Sun et al. [12]; Kaine et al. [13]; Kampe et al. [14]; Lee et al. [15]

In the present meta-analysis, seven studies were included (773761 patients and 79184 events), five studies were retrospective and two were cross-sectional. Diabetes was common among patients with psoriasis compared to control subjects; odds ratio: 1.38, 95% CI: 1.17-1.64, P-value = 0.0002, chi-square = 224.93; and standard difference = 6. However, substantial heterogeneity was found, *I^2^* For heterogeneity, 97%, and P-value <0.001; therefore, the random effect was applied (Figure 2).

**Figure 3:**
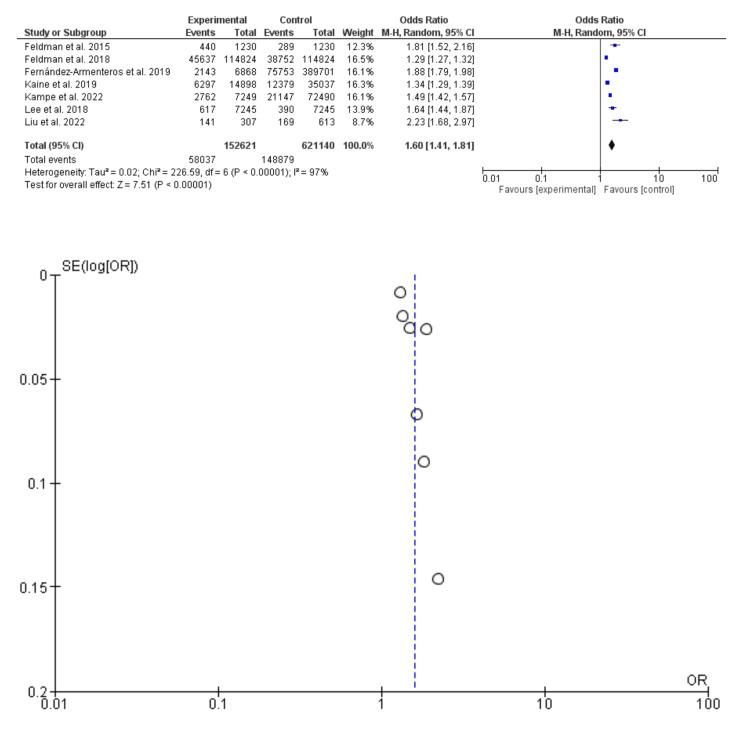
Hypertension Among Patients With Psoriasis and Control Subjects Feldman et al. [9]; Fernández-Armenteros et al. [10]; Feldman et al. [11]; Sun et al. [12]; Kaine et al. [13]; Kampe et al. [14]; Lee et al. [15]

Hypertension was more common among patients with psoriasis compared to their counterparts; odds ratio 1.60, 95% CI: 1.41-1.81, P-value = 0.00001, chi-square = 226.59, and standard difference = 6. However, substantial heterogeneity was found,* I^2^* for heterogeneity, 97%, and P-value <0.001. The random effect was used due to the substantial heterogeneity (Figure 3).

Discussion

First and foremost, numerous epidemiological studies have consistently demonstrated a strong association between psoriasis and diabetes mellitus. A systematic review and meta-analysis published in the Journal of the American Academy of Dermatology in 2012 found that individuals with psoriasis had a significantly increased risk of developing diabetes compared to those without the skin condition [8].

In addition to the link between psoriasis and diabetes, a growing body of evidence has also implicated hypertension as a comorbidity of psoriasis. A systematic review and meta-analysis found that individuals with psoriasis had a significantly higher prevalence of hypertension compared to those without the skin condition. This association persisted across different age groups and was independent of traditional cardiovascular risk factors. Moreover, a large-scale cohort study conducted in the United States demonstrated that the risk of developing hypertension was significantly elevated in patients with severe psoriasis, further underscoring the need for comprehensive cardiovascular risk assessment in this population [16]. A few other meta-analyses assessed the relationship between psoriasis and hypertension and found an association between mild and severe psoriasis and high blood pressure, confirming the findings of another meta-analysis [16-17]. Plausible explanations might include stress, anxiety, depression, psoriasis drugs, and a sedentary lifestyle among patients with psoriasis [18]. In addition, psoriasis is a systemic inflammatory disorder associated with other comorbidities, including obesity and diabetes, which might increase the risk of high blood pressure [19]. The previous study was limited by many confounders that might affect their conclusion, emphasizing the importance of adjusting for confounders to avoid the risk of bias and heterogeneity [20]. Furthermore, the relationship between diabetes mellitus and hypertension has been extensively studied, with evidence suggesting a bidirectional association between the two conditions. A systematic review and meta-analysis published in Diabetes Care in 2011 found that individuals with diabetes had a significantly higher risk of developing hypertension compared to those without diabetes. Conversely, individuals with hypertension were found to have a significantly increased risk of developing diabetes. These findings underscore the intricate interplay between diabetes and hypertension and the need for integrated management strategies to mitigate the risk of cardiovascular complications in affected individuals [20].

Screening for hypertension among patients with psoriasis is vital to avoid serious complications, including myocardial infarction, stroke, and renal complications [21]. In the present study, we found that patients with psoriasis were more likely to have diabetes compared to their counterparts without the disease, which is in line with another systematic review and meta-analysis [22], which included five studies and observed similar findings.

Systemic inflammation and adipocytokine dysregulation increase insulin resistance and the progression to type 2 diabetes [4]. Importantly, etanercept therapy was found to reduce fasting insulin and improve insulin sensitivity [23], an effect that was not observed when using PUVA (psoralen plus ultraviolet-A) therapy [4]. These findings imply that biological therapy and inflammation prevention are real paradigm shifts in optimizing the treatment of patients with psoriasis to improve outcomes.

Another meta-analysis found shared four genetic loci between diabetes and psoriasis, suggesting a causal relationship [24]. Low levels of adiponectin and high levels of fetuin-A were observed among patients with psoriasis [25], with high levels of fetuin-A suggested to increase insulin resistance and keratinocyte proliferation, enhancing skin inflammation and the development of metabolic syndrome [26]. Interestingly, genetically predicted glycated hemoglobin (HbA1c) increases the risk of psoriatic arthritis, and genetic liability to both psoriasis and psoriatic arthritis increases the risk of cardiometabolic disease, including coronary artery disease [27]. Screening for cardiovascular and metabolic disorders among patients with psoriasis is important for timely management to reduce mortality and improve the quality of life [28].

The association of diabetes mellitus and psoriasis has important therapeutic implications. A lower level of low-density lipoproteins is recommended, as most statins might increase the incidence of newly diagnosed diabetes. In addition, psoriasis is associated with dyslipidemia. Therefore, choosing a lipid-lowering drug with a low risk of diabetes (including pravastatin and pitavastatin) is important [29].

The limitations of this study were the small number of included studies, the observational nature of the studies, and the significant heterogeneity observed.

## Conclusions

There is clear evidence of an association between psoriasis, hypertension, and diabetes mellitus, with psoriasis often preceding the development of these comorbid conditions. Patients with psoriasis have a higher chance of developing diabetes mellitus; in addition, hypertension was more common among patients with psoriasis compared to their counterparts without the disease. The substantial heterogeneity observed limited the current conclusion.

Individuals with psoriasis should be aware of these potential risks and work with their healthcare providers to monitor and manage their overall health. Randomized controlled trials are needed to assess the best therapeutic approach for psoriasis and its associated cardiovascular risk factors.
